# BamClassifier: a machine learning method for assessing iron deficiency

**DOI:** 10.1038/s41598-025-92892-y

**Published:** 2025-09-02

**Authors:** Emmanuel S. Adabor, Patrick Adu, Daniel Adomako Asamoah

**Affiliations:** 1 School of Technology, Ghana Institute of Management and Public and Administration, Accra, Ghana; 2 Department of Medical Laboratory Science, School of Allied Health Sciences, University of Cape Coast, Cape Coast, Ghana; 3 Wright State University, Dayton, OH USA

**Keywords:** Iron deficiency, Iron deficiency assessment, Machine learning, Classification, Machine learning, Computer science

## Abstract

Iron deficiency (ID) is a well-known cause of anaemia and could lead to adverse clinical and functional impairments. However, ID is under-diagnosed due to non-specific symptoms, difficulties in interpreting ambiguous assessment outcomes and suboptimal sensitivities of methods in some circumstances. In this study, we present BamClassifier, a machine learning method for assessment of ID. This method proceeds by repeated selection of samples of instances from routine complete blood count data in such a way that each observation is included in exactly one sample. Then, a median-supplement machine learning model built from each sample, and the performance of the model on test instances are aggregated into a bag of predictions from which ID statuses are assigned to samples by way of the highest frequency counts. We show the effectiveness of our method by applying to real datasets obtained from different investigations in Ghana and simulated data as well. Our method obtained perfect area under receiver operating characteristic curve in all experiments and significantly outperformed other well-established methods in terms of accuracy, sensitivity, specificity, precision, and diagnostic odds ratio in all our evaluations. A successful application of the method will permit the study of large collections of samples for ID assessments, save time and cost using complete blood count parameters, while standardizing interpretation of outcomes of such investigations.

**Supplementary Information:**

The online version contains supplementary material available at 10.1038/s41598-025-92892-y.

## Introduction

Iron deficiency (ID) is a widespread nutritional problem affecting millions of people worldwide^1^. It is one of the leading causes of diseases affecting children and premenopausal women globally, and people living in low- and middle- income countries^2^. Besides being well known to be the frequent cause of anaemia, ID could lead to certain clinical and functional impairments^3^. Early detection and accurate assessment of iron deficiency are crucial for effective intervention and prevention strategies. Individuals affected by ID experiences suboptimal physical performance, work productivity, and cognitive function^4^. Nevertheless, ID is under-diagnosed for various reasons suggested in earlier studies^5,6^. This is despite the methods proposed for its assessments. For instance, the use of Haemaglobin (Hb) alone for assessing ID is associated with low sensitivity and specificity requiring further standardization^7,8^. Another method characterized by low specificity is the use of mean corpuscular volume (MCV) and reticulocyte Hb contents^9^. It has been reported that an increased MCV could result in false normal values of the reticulocyte Hb contents^10^. Besides these, other methods have been noted to have high sensitivity, which are not widely adopted.

Erythrocyte zinc protoporphyrin (ZnPP) is one of such methods. Its values are increased by chronic infections, inflammations and other diseases leading to low specificity^11,12^. Thus, adopting ZnPP in routine clinical use will require a time-consuming process as well as a need to standardize cut-off for ZnPP in using it to assess ID given the varied specifications in the literature^13–15^. On the other hand, the use of transferrin saturation for assessing ID based on plasma iron and iron-binding ratio is a widely adopted method. However, it has been criticized because of the effects of other disorders on transferrin levels^16^. The use of serum ferritin (SF) method for assessing ID has been shown to be a better performer compared to the other methods such as the MCV, ZnPP, and transferrin saturation^17^. Nevertheless, as levels of serum ferritin are increased above normal values by chronic inflammation, its estimates could be misleading in the presence of liver and other related diseases, and in a case where there is high alcohol intake^10^. In other instances, the serum transferrin receptor (TfR) has been employed in assessing iron-deficient anaemia (IDA). Nonetheless, its wide applicability is limited by high cost, lack of standardized values and difficulties in accessing such data values in children^10^. The ratio of serum transferrin receptor to serum ferritin (TfR/SF) approach for assessing ID has been proposed. Even though this is effective in elderly subjects, it is adversely affected by the lack of standardization of TfR assays^10^. There have been other approaches to improve ID assessments by combining several indices, but they lead to trade-offs between sensitivity and specificity^18^. Currently, the bone-marrow biopsy is the gold standard method for assessing ID when performed under standardized conditions. However, it requires an expert to examine the marrow. Technical experts are uncommon and the method is uncomfortable to subjects, making them non-routine practice in some settings^10^. These have necessitated the introduction of non-invasive methods such as computational technologies.

In recent years, computational methods have emerged as valuable tools for assessing iron deficiency, offering non-invasive and cost-effective alternatives to traditional diagnostic approaches. Kurstjens and coworkers used a random forest model to assess the risk of low ferritin levels using minimal complete blood count (CBC) and C-reactive protein (CRP)^19^. Although this would help to support diagnosis of otherwise unrecognized iron deficient anaemia, a significant number of machine learning tools have mainly depended on the usage of radiological and other related images as different features in predicting iron deficient anaemia^20^. However, other methods, such as the neural network were used to achieve a similar goal using four features from accessible laboratory data^21^. The need to advance the developments of computational methods for assessing ID is driven by the success of these earlier applications, and the need to find the true ID devoid of effects of other sources of iron deficient erythropoiesis.

In this study, we present a novel machine learning method for assessing true ID based on serum ferritin, CRP, and CBC parameters, and demonstrate its application along with some other machine learning methods for determining ID based on routine CBC data. The new method is founded on the ideas of MSclassifier models^22,23^ and the principle of reducing variance of statistical learning. Particularly, it involves selecting subsamples from the data in such a way that each observation is included in exactly one sample and a combination or union of all subsamples make up the entire observations, building MSclassifier model on each sample, and aggregating the performance of each model to make predictions about ID statuses of instances. In this way of bagging model predictions, this new method, Bamclassifier, exploits the property of enhancing datasets for effective knowledge discovery while modelling the underlying assumptions that accompany the measured laboratory attributes of ID. This is due to the probabilistic nature of the modelling that best fits random experiments. While these features distinguish the new method, our motivation for proposing a more efficient method is based on the results of previous studies^22^. Particularly, it was shown that median-supplement machine learning approaches outperformed other bagging approaches such as random forest in binary classification task in breast cancer^22^. Furthermore, data is obtained from laboratory measurements of apparently healthy individuals in Ghana. Real datasets were used to test the method and hence, makes it suitable for applying it to diagnose ID. Moreover, additional simulated datasets are simulated and used to assess the stability and robustness of the new method.

## Materials and methods

### Data sets

Two different types of real datasets were obtained for the study. The first dataset was obtained from a baseline FBC-ferritin of prospective blood donors across three geographic regions in Ghana namely Northern, Eastern and Central^24^. However, the variables that were reported in the Eastern region’s data were fewer and not consistent with the datasets from the other two regions. In particular, data from the Northern and Central Regions had information about Age, Gender, WBC, HB, MCV, MCH, MCHC, PLT, HCT, CRP, and ferritin. Data from the Eastern region had only HB, MCV and MCH and so it was not included in the analysis. The variables such as MCV, MCH, MCHC, HB, and RBC were selected because they have been used in an artificial neural network model to predict the presence or absence of IDA in the past^21^. The attributes considered in this study are directly or indirectly associated with ID.

We excluded instances with CRP > 5 mg/l as it gives an indication of confounding effects of inflammation on ferritin^25^. This and other instances of incomplete records of samples reduced an initial sample of size 190 to 188 as available set of instances in the first dataset after preprocessing. The second dataset was obtained from nulliparous women aged between 16 and 36 years with no clinically diagnosed condition from two zonal divisions in Ghana. This dataset comprised 336 instances. This data was collected as part of a study that assessed the iron stores in preconception nulliparous women in peri-urban Ghana^26^. The data comprised of AGE, BMI, WHR, HB, MCV, MCH, MCHC, CRP, and serum ferritin (SF) measurements. The presence of iron deficiency (referred to as ID positive status) was defined as SF < 15µ g/l^10^. Those instances which are not iron deficient are referred to as ID negative in this study. Based on the criterion defined in^10^, the first dataset comprised 34 ID positive and 154 ID negative statuses, while the second dataset had 109 ID positive and 207 ID negative statuses. Datasets used for the analysis and assessment of methods used for this study are included in this manuscript as supplementary materials.

The Institutional Review Board of the University of Cape Coast approved all protocols for the studies on which this study was based (ethical clearance ID: UCCIRB/CHAS/2016/46). The study protocols conformed to the provisions of Helsinki declaration including confidentiality, risks and benefits assessments, consent to participate, and ensuring respect to participants. Participants read and signed written informed consent before being enrolled on the respective study. Participants were also made aware that they could withdraw from the study at any point in time and their medical records would be kept and treated with strict confidentiality.

### BamClassifier

**Fig. 1:**
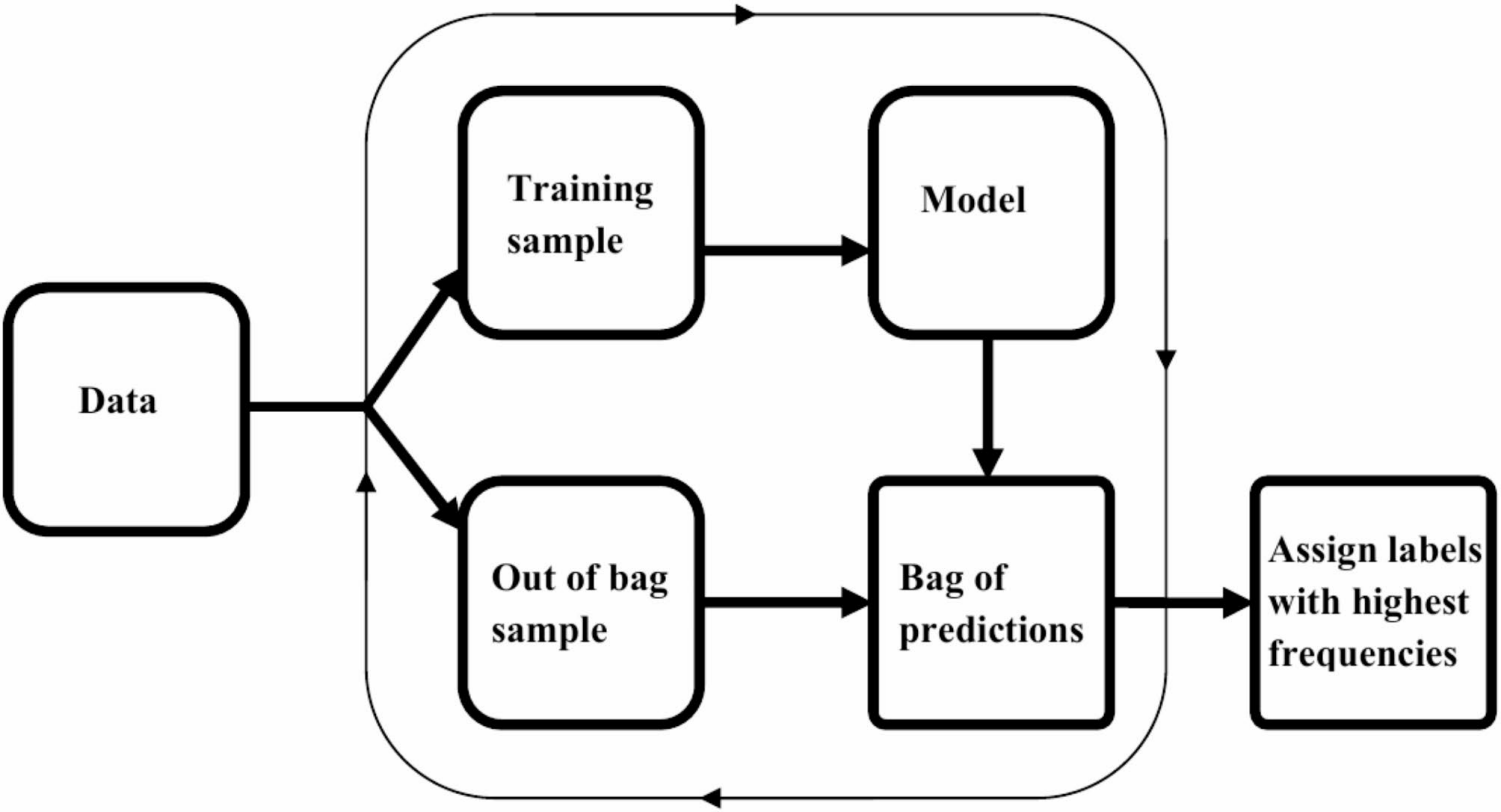
Overview of BamClassifier. A subsampling of equally sized samples is selected repeatedly without replacement. Each subsample is used to develop a median-supplement naive Bayes model and tested on the unused samples (out-of-bag samples). The predictions of the out-of-bag samples are aggregated into a consensus bag, bag of predictions, where labels for each unique instance is assigned the majority class or label among its predictions.

Machine learning methods have been found useful for several classification tasks in precision medicine. The need for enhanced methods emerges from aspirations to obtain better performances of methods, allow methods to incorporate the dynamic features of variables/attribute measurements and effectively handle complex biological systems. In the case of assessing the ID statuses, we introduce a new method that reduces learning variance by bagging and supplements disparities in ID positive and negative instances using median-supplement machine learning methods to improve predictive accuracy of the new method. An overview of the approach is presented in Fig. 1.

### Sub-sampling for training and testing model

For a given data with *n* instances, a sub-sampling of approximately equally sized samples is randomly selected repeatedly without replacement from the original data. This sampling continues until every instance in the original set has been included in a subsample. For each subsample, a classifier is developed and tested with the out-of-bag samples. This sampling technique allows every instance to be included in developing models, and as well as testing the model at some point in the process. Here, we let *B* denote the number of sub-samples that are drawn from the original data made up of *n* instances. The predictions of the out-of-bag samples are aggregated into a consensus bag, referred to as bag of predictions. In the bag of predictions, the label for each instance is the majority class or label.

### Building a bamclassifier

For every subsample, a median-supplement naive Bayes classifier^22^ was developed as the learning model. Here, it is incorporated in the models for assessing iron deficiency based on serum ferritin levels. The difference between instances of the ID positive and ID negative statuses is determined. Let this difference be *m*. With this determined, an *m by k* matrix of Latin Hypercube^27^ with uniformly distributed probability values are generated for *k* features of the selected subsample. In this way, each column of the matrix will correspond to the features of the samples. A scalar multiplication of the median of each feature and corresponding column (vector) of the Latin Hypercube is determined. That is, suppose $x{}_{i}$ represents the vector of i-th attribute values in a sample, and $x_{i}^{\prime }$ is the corresponding column in the *m by k* matrix, the supplementary data corresponding to the i-th attribute, denoted by $s_{i}$ will be given by Eq. (1):

1 $${s_{i} = med\left( {x_{i} } \right)x_{i}^{\prime } },$$

where ${med\left( {x_{i} } \right)}$ is the median of the i-th attribute values in a sample. These supplementary observations corresponding to i-th attribute is included in the training set as additional sets of instances for attribute *i*. That is, if there were *j* observations for the attribute *i* in the training set, it will be extended to (*j + m)* observations such that the labels of the new instances will be the lesser label of the original instances. For example, suppose for attribute *i*, there are 10 samples with 4 ID positive and 6 ID negative statuses in training set. Then, the difference between the numbers of statuses, *m*, is 2 (6 minus 4), and two additional instances with the ID positive labels will be added to the 10 instances to make it 12. The values of the attributes for the two additional (supplementary) instances is given by scalar multiplication of the median of attribute *i* and the column vector corresponding to the *i-th* attribute of the *m* by *k* matrix.

Applying this to every column of the *m* by *k* matrix results in the transformation of the *m* by *k* matrix which becomes the supplementary data included in the subsample from which the classifier is inferred. This balances the differences between the ID statuses to equal numbers of instances to improve the accuracy of every model built from each subsample as the supplementary data will bear the label of the lesser ID status in the original subsample. Every model inferred from the subsamples by this approach improves its effectiveness in predicting the out-of-bag samples. The aggregation of all the predictions is stored in a bag of predictions where labels are assigned to every test instance based on the number of consensus predictions. For every instance, the label with the highest frequency among its predicted labels is assigned to it. The steps involved in building BamClassifier are outlined below:

1. Specify the size of subsamples or folds for model building.
2. Sample a subsample from the initial set without replacement.
3. Build a median-supplement naive Bayes model on the selected sample.
4. Predict the out-of-bag sample instances (i.e., predict every other instance).
5. Repeat steps 2 to 4 until every instance has been included in exactly one sample for model building in step 3.
6. Aggregate all predictions in step 4 into a consensus bag, called bag of predictions.
7. For each instance, assign the most probable label, which is indicated by the label with the highest frequency.

### Naive Bayes method

Using the Bayes theorem, it can be shown that for a given set of features, *G*, corresponding to several instances of ID statuses, the probability that any ID assessment is classified into a single ID status, say positive (${C_{{ + ve}} }$), is given by Eq. (2):

2 $${P\left( {C_{{ + ve}} |G} \right) = \frac{{P\left( {G|C_{{ + ve}} } \right)P\left( {C_{{ + ve}} } \right)}}{{P\left( G \right)}}}$$

where ${P\left( {G|C_{{ + ve}} } \right)}$ is probability of instance *G*, given that the sample ID status is positive, ${P\left( {C_{{ + ve}} } \right)}$ is the probability that ID status is positive, and *P*(*G*) is the probability of observing the set of features *G*. In this way, the ID status of any sample is determined to be positive if this class has the highest posterior probability. However, it is assigned negative if the negative class has the highest posterior probability. This approach to classification assumes that distribution of the effective variables is independent, and this is consistent with the current measured attributes ID statuses’ data. This suggests that the conditional probability on the right-hand side of Eq. (2) can be expressed as Eq. (3) for any i-th feature of *k* features, ${g_{i} }$, under consideration in the ID assessment problem.

3 $${P\left( {G|C_{{ + ve}} } \right) = P\left( {g_{1} |C_{{ + ve}} } \right)P\left( {g_{2} |C_{{ + ve}} } \right) \ldots P\left( {g_{k} |C_{{ + ve}} } \right) = \prod P \left( {g_{k} |C_{{ + ve}} } \right)}$$

### Performance measures

For benchmarking, the proposed method is compared to other well-established best performing probabilistic and tree-based methods namely naive Bayes, Bayesian networks, logistic regression, random trees and random forest methods implemented in Weka^28^. These methods are compared on the basis of performance indicators which are true positive, true negative, false positive and false negative. When an instance of ID positive status is correctly predicted as positive, it is counted as true positive (TP). When an instance of ID positive status is wrongly predicted as negative, it is counted as false negative (FN). When an instance of ID negative status is correctly predicted as negative, it is counted as true negative (TN). When an instance of ID negative status is wrongly predicted as positive, it is counted as a false positive (FP). These observations are assessed in cross-validation used to evaluate every method in this study. While a 4-fold is used for evaluation of methods on actual datasets, a 10-fold cross-validation is used for the simulated datasets. The use of cross-validation reduces the likelihood of overfitting by the various methods used in this study.

With these indicators, the abilities for the methods are evaluated using the following measures:

4 $$Accuracy = \frac{{TP + TN}}{{TP + TN + FP + FN}},$$

5 $$Specificity = \frac{{TN}}{{TN + FP}},$$

6 $$Sensitivity = \frac{{TP}}{{TP + FN}},$$

7 $$Pr ecision = \frac{{TP}}{{TP + FP}},$$

8 $${Diagnostic\,odds\,ratio\,\left( {DOR} \right) = \frac{{TP \times TN}}{{FP \times FN}}}$$

The maximum value for the metrics accuracy, precision, specificity, and sensitivity is 100% and a good model approaches this maximum value as much as is possible. The accuracy measures the classification rates for each method in assessing ID status for any sample. While sensitivity measures the methods’ performances on predicting ID positive instances, specificity indicates the performance on correctly distinguishing instances that are not ID positive status. Precision assesses the predictive performances relative to the false positive counts. On the other hand, the diagnostic odds ratio (DOR), representing the ratio of the product of correct predictions to false predictions, is expected to be greater than one (1) for good models although it is also desirable to have large value.

In the overall assessments of how well a method performs, the trade-off between sensitivity and false positive rate (1 – specificity) are visualized in the receiver operating characteristics (ROC) curve^29,30^. The area under the ROC curve (AUC) further provides an indication of how well a model performs indicating discriminating power in deciphering different ID statuses as has been shown in machine learning applications in the past^31,32^. For any model, the AUC can reach a maximum of 100%. The higher the AUC, the better the model. Good models are expected to have a higher AUC compared to any random model. The proposed method and its analysis were implemented in the R statistical software version 4.2.2.

### Statistical tests

Analysis of data to assess normality and to compare equality of means of ferritin levels between groups of samples were conducted in R. Shapiro test was used to verify normality of the data. The data was not found to be normally distributed (*p* < 0.001). Therefore, a Mann-Whitney test was used to compare differences between groups of samples. All statistical testing of significance was performed at 5% level of significance.

### Assessing the stability of performance of BamClassifier

In order to assess stability of the model’s performance, 100 bootstrap samples were generated from the original dataset. The model was trained on each sample and tested on the corresponding out-of-bag (OOB) data. Performance metrics were recorded for each iteration, and the means, standard deviations, and 95% confidence intervals were calculated to assess the model’s stability. Additionally, a line plot was used to visualize performance metrics across the bootstrap iterations, illustrating the trends and consistency of the model’s performance over time.

### Robust assessment of BamClassifier

We conducted robust assessment of the proposed method. In doing this, we assessed its performance by varying factors such as class imbalance, feature noise, and data distribution. The original datasets were not found to follow any known standard distribution. Therefore, an empirical distribution was assumed as an estimate of the underlying distribution of each attribute or feature^29^. This enabled us to sample 1000 instances from each distribution. Imbalanced labels (90:10) were randomly assigned to the instances, and the model was evaluated to observe its performance on imbalanced data. In order to assess the effect of feature noise, we simultaneously introduced noise of varying levels into two features in two experiments, and half of the features in another experiment. Details are provided in the supplementary material with implementation code. In order to further vary data distributions, we sampled 1000 samples for each feature from a Gamma distribution with shape and rate parameters aligned with the original features to approximate the underlying characteristics. Details of these robust assessments are provided in the supplementary material with implementation code. For each robust assessment, a 10-fold cross-validation was performed on datasets of 1000 samples.

###  Code availability

All computer codes supporting this study are available as supplementary material.

## Results

### Data summary

As discussed in the methodology, both real and simulated datasets are used in the evaluation of the proposed method, BamClassifier. The performances of BamClassifier on the datasets collected for the study assessing ID statuses were compared with the performances of well-performing established methods including random forest, naïve Bayes, Bayesian networks, logistic regression, and random trees. A summary of the data is presented in Table 1.

**Table 1:** Summary statistics of actual data.

Variable	Median	Mean	Standard deviation
Both males and females data			
Total, n	188		
Gender, %male, %female	86%, 14%		
WBC (×10^9^/L)	5.21	5.62	3.42
RBC (×10^12^/L)	5	5.07	0.62
Hgb (g/dL)	14.9	15.08	2.05
MCV (fL)	80.5	79.88	7.12
MCH (pg)	29.9	30.09	5.02
MCHC (g/L)	36.6	39.18	29.57
PLT (×10^9^/L)	210	213.01	75.63
HCT (L/L)	40.15	40.57	4.47
Nulliparaous women data			
Total, n	316		
BMI (kg/m2)	23.08	23.62	3.5
WHR	0.77	0.78	0.05
HB (g/dl)	11.7	11.64	1.78
MCV (fL)	84	86.29	12.37
MCH (pg)	28.45	30.83	27.21
MCHC (g/L)	33.3	33.99	31.24

### Ferritin levels differed among male and female samples

**Fig. 2:**
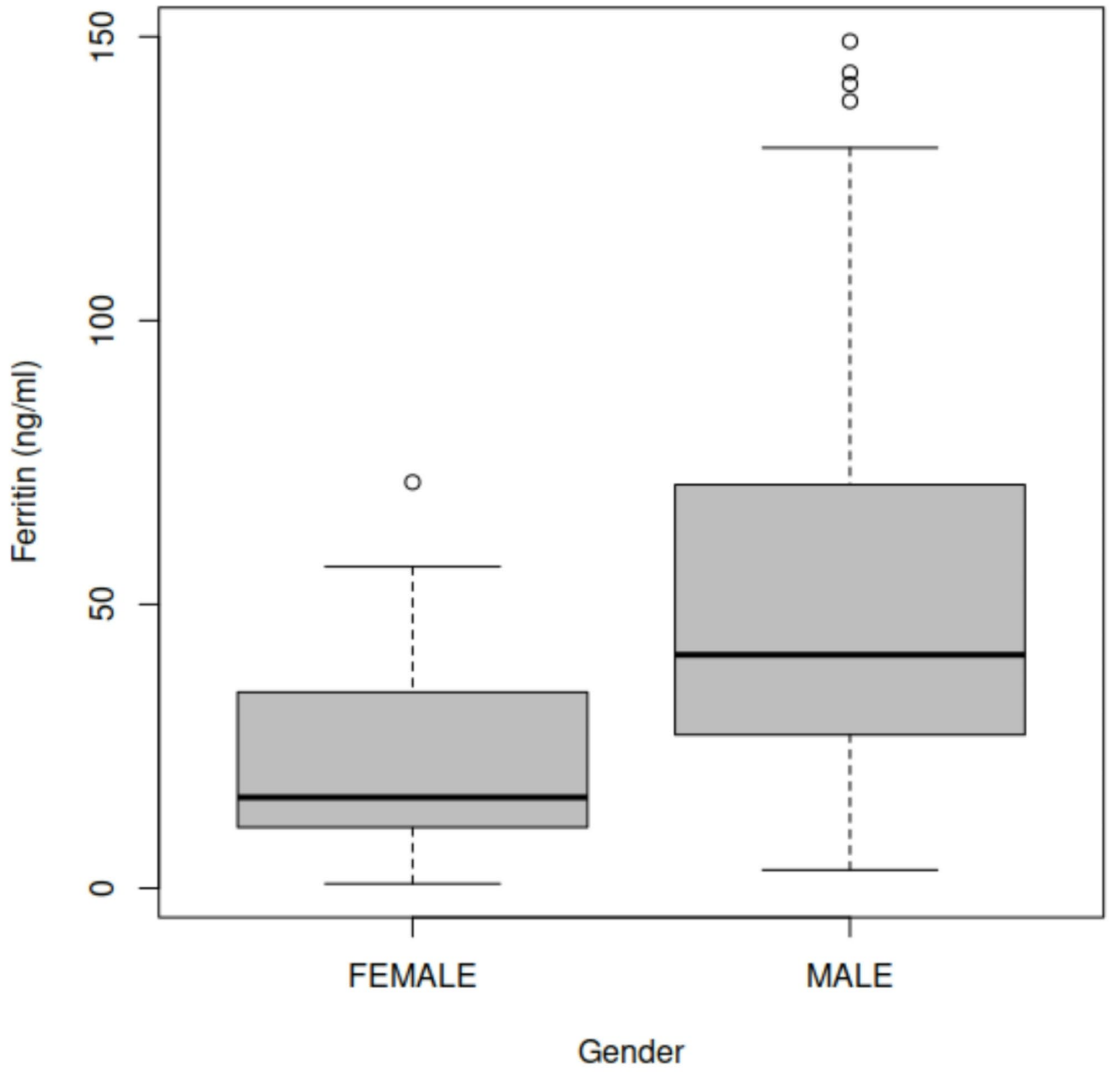
Variability of ferritin levels in female and male participants of the study.

The first dataset comprised of samples obtained from 161 males and 27 females. It was found that the ferritin levels varied in most cases with sex (Fig. 2). The ferritin levels in males were found to be higher compared to female participants of the study (Fig. 2). A further investigation of these differences between ferritin levels of the female and male groups was found to be statistically significant (*p* < 0.001). These results were to be expected since total body iron content in men averages approximately 50 mg/kg compared to 35 mg/kg in women^33^.

### BamClassifier accurately assesses iron deficiency

**Fig. 3:**
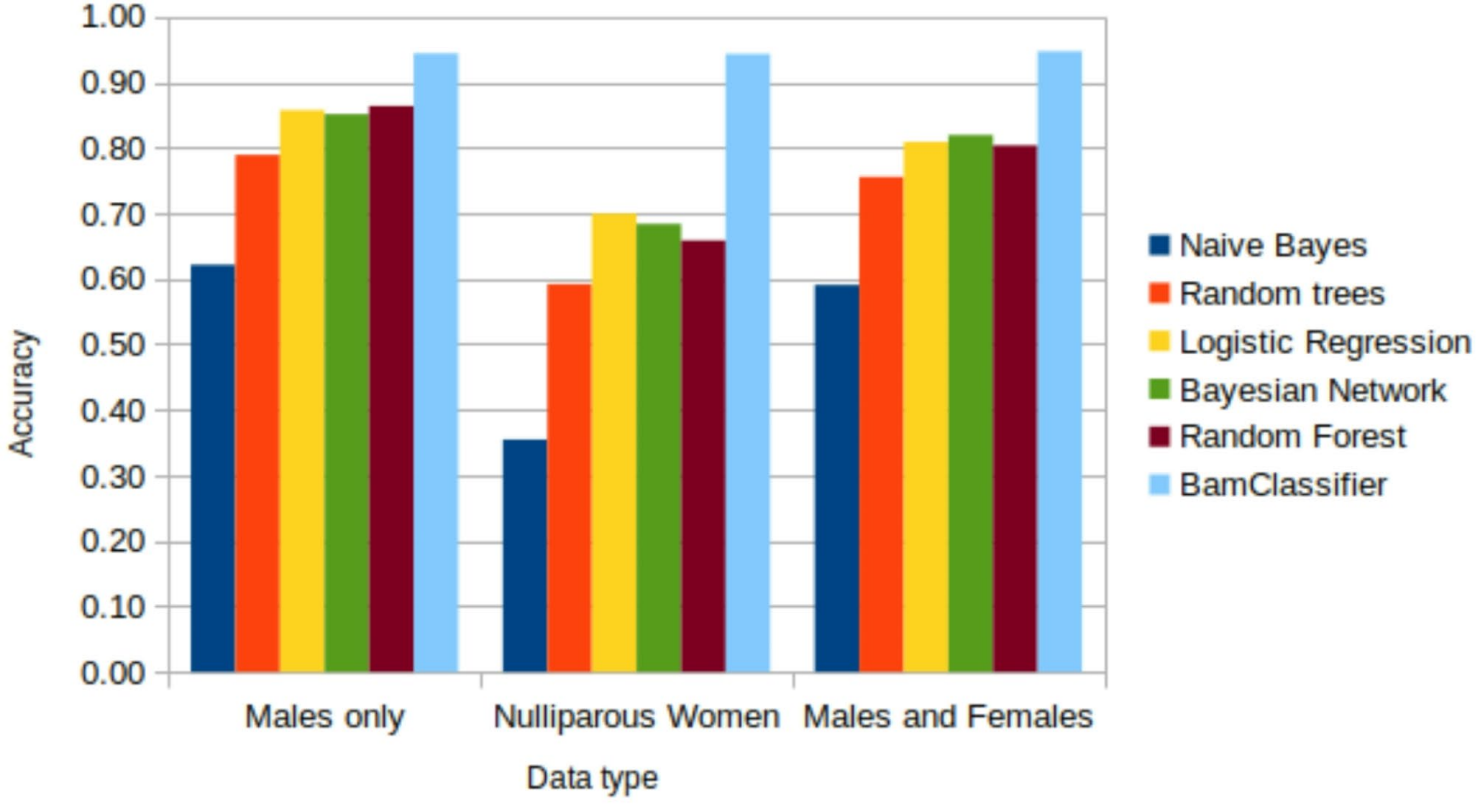
Accuracy of BamClassifier compared to Naive Bayes, Random trees, Logistic Regression, Bayesian Network and Random Forest. BamClassifier outperforms all methods on Males’ only, nulliparous women and both males and females’ groups. Results are produced from 4-fold cross-validation.

Due to the statistically significant difference between the groups of participants, the proposed model was applied to the different groups and then to the combined groups. On the application of the BamClassifier to males’ only group, it outperformed all the other established methods in assessing the ID as it obtained the best accuracy (Fig. 3).

### BamClassifier exceeds expectations on performance metrics

Besides obtaining the best accuracy, BamClassifier also demonstrated superiority in identifying ID among apparently healthy individuals by achieving the highest sensitivity, precision, and Diagnostic Odds Ratio (DOR) among all the machine learning methods employed in this study (Table 2). All the methods considered in this study achieved higher performance on distinguishing non-iron deficient samples as they obtained specificity that ranged between 87% and 99% except the naive Bayes method, which had 63% (Table 2). These high performances were to be expected because most (86%) of the training instances of the males’ only data had ID negative statuses. Machine learning methods such as those presented in this study improve performance with increasing sizes of training set^34^. Besides, it has been shown that increasing differences in labels of binary classification problems increases the performance of median-supplement machine learning methods^22^.

**Table 2:** Performance of machine learning methods on males’ only data.

	Sensitivity [95% CI*]	Precision [95% CI]	Specificity [95% CI]	DOR** [95% CI]
Naive Bayes	0.59 [0.51, 0.66]	0.20 [0.14, 0.26]	0.63 [0.56,0.70]	2.42 [0.97, 6.04]
Random trees	0.27 [020, 0.34]	0.25 [0.18, 0.32]	0.87 [0.82, 0.92]	2.52 [0.87, 7.28]
Logistic Regression	0.18 [0.12, 0.24]	0.44 [0.36, 0.52]	0.96 [0.93, 0.99]	5.96 [1.46, 24.24]
Bayesian Network	0.05 [0.02, 0.08]	0.25 [0.18, 0.32]	0.98 [0.96, 1.00]	2.16 [0.21,21.73]
Random Forest	0.09 [0.05, 0.13]	0.50 [0.42, 0.58]	0.99 [0.97,1.00]	6.85 [0.91, 51.40]
BamClassifier	0.86 [0.81, 0.91]	0.76 [0.69, 0.83]	0.96[0.93,0.99]	139.33 [32.13, 604.18]

*CI is confidence interval.

**DOR is diagnostic odds ratio.

Furthermore, although we found the naive Bayes method less likely to make correct predictions (DOR < 1), it was able to predict 89.91% of ID positive statuses correctly among the nulliparous women (Table 3). This accounted for it achieving the 1% higher sensitivity than BamClassifier. However, the low value of the DOR was due to relatively large numbers of incorrect predictions observed in the false negative and positive counts. Nevertheless, the BamClassifier achieved the best performance in terms of precision, specificity and DOR when applied to the nulliparous women data (Table 3). The high values of DOR achieved by BamClassifier indicate that it produces the highest correct predictions compared to the other methods. It is worthy of note that BamClassifier achieved specificity and precision as high as 97% and 94% respectively. However, naive Bayes method could only achieve 7% specificity and 34% precision on the nulliparous women data making it undesirable ID assessment method involving such kind of data. The superiority of BamClassifier is further demonstrated in the reported accuracy measures. The BamClassifier significantly exceeded the accuracy of the other methods (Fig. 3). Interestingly, the results suggest that although the methods could have improved performance on smaller dataset, BamClassifier has the best performance on both smaller and larger sized datasets as seen in both the male’s only and the nulliparous women data.

**Table 3:** Performance of machine learning methods on nulliparous women data.

	Sensitivity [95% CI*]	Precision [95% CI]	Specificity [95% CI]	DOR** [95% CI]
Naive Bayes	0.90 [0.87,0.93]	0.34 [0.29, 0.39]	0.07 [0.04, 0.10]	0.65 [0.28, 1.48]
Random trees	0.36 [0.31,0.41]	0.40 [0.35, 0.45]	0.71 [0.66, 0.76]	1.40 [0.85, 2.29]
Logistic Regression	0.31 [0.26, 0.36]	0.63 [0.58, 0.68]	0.90 [0.87, 0.93]	4.24 [2.29, 7.83]
Bayesian Network	0.14 [0.10, 0.18]	0.71 [0.66, 0.76]	0.97 [0.95, 0.99]	5.35 [2.01, 14.21]
Random Forest	0.33 [0.28, 0.38]	0.51 [0.45, 0.57]	0.83 [0.79, 0.87]	2.42 [1.41, 4.16]
BamClassifier	0.89 [0.86, 0.92]	0.94 [0.91, 0.97]	0.97 [0.95, 0.99]	268.00 [97.64, 735.61]

*CI is confidence interval.

**DOR is diagnostic odds ratio.

In a further analysis, we considered the first data as was collected from the field by applying the methods to all the 188 instances (of males and females). In this experimentation, we did not find profound differences in the levels of performance of the methods from the experiments with the other datasets. However, the Bayesian Network method was not able to predict any of the ID positive statuses although it did not fail to identify any of the ID negative instances. This resulted in its ability to achieve a 100% specificity, while no sensitivity, precision or DOR was achieved (Table 4). Besides the measure of specificity in which Logistic Regression method and BamClassifier performed equally highly (97%), BamClassifier was the best performer in terms of sensitivity and DOR (Table 4).

**Table 4:** Performance of methods on both males and females’ data.

	Sensitivity [95% CI*]	Precision [95% CI]	Specificity [95% CI]	DOR** [95% CI]
Naive Bayes	0.65 [0.58, 0.72]	0.25 [0.19, 0.31]	0.58 [0.51, 0.65]	2.51 [1.16, 5.44]
Random trees	0.35 [0.28, 0.42]	0.33 [0.26, 0.40]	0.84 [0.79, 0.89]	2.95 [1.29, 6.76]
Logistic Regression	0.09 [0.05, 0.13]	0.38 [0.31, 0.45]	0.97 [0.95, 0.99]	2.88 [0.65, 12.70]
Bayesian Network	0.00	-	1	-
Random Forest	0.09 [0.05, 0.13]	0.33 [0.26, 0.40]	0.96 [0.93, 0.99]	2.39 [0.57, 10.06]
BamClassifier	0.85 [0.80, 0.90]	0.85 [0.80, 0.90]	0.97 [0.95, 0.99]	172.84 [47.02, 635.39]

*CI is confidence interval.

**DOR is diagnostic odds ratio.

**Fig. 4:**
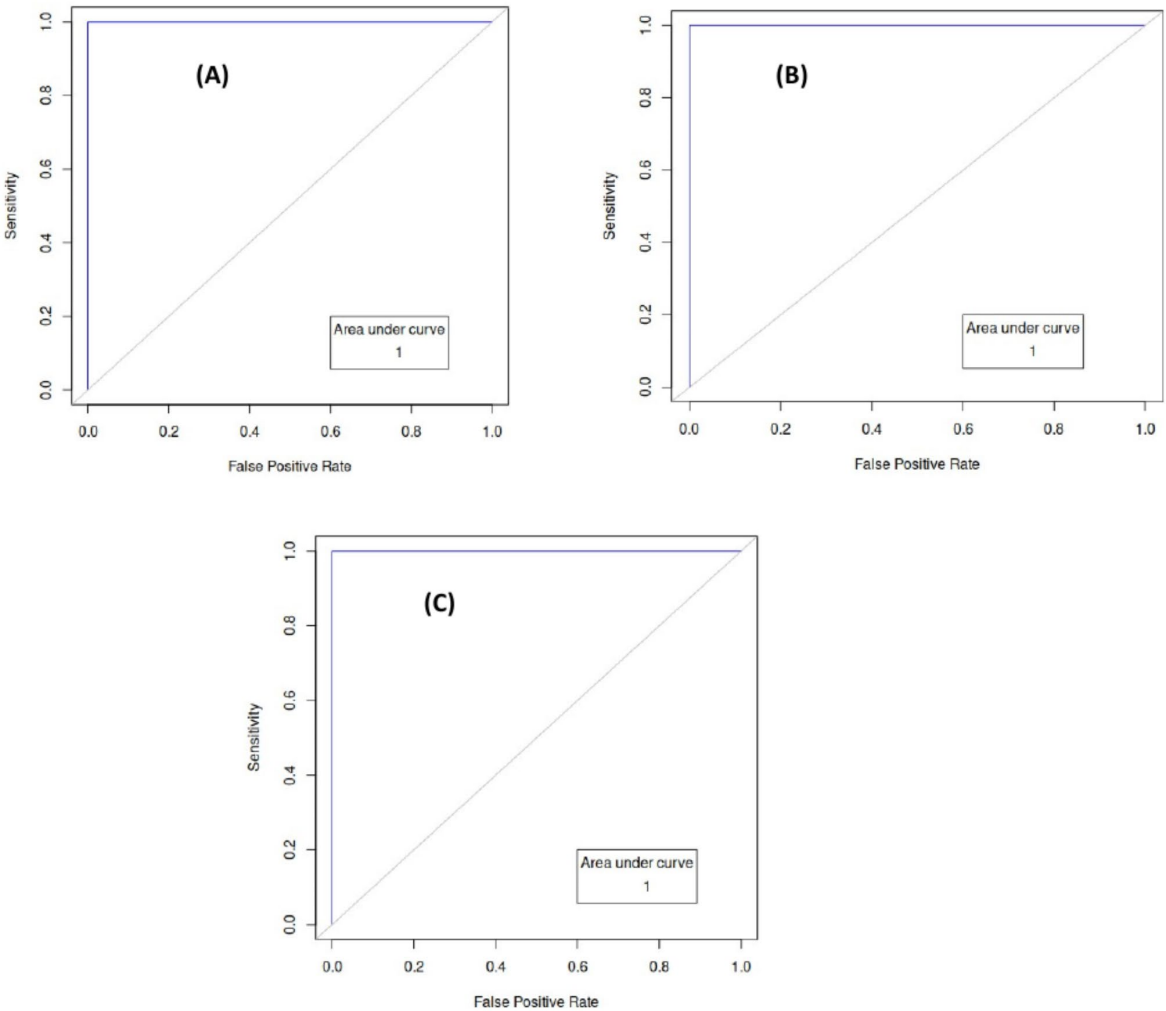
Receiver-operating characteristic (ROC) curve for BamClassifier. ROC curves for BamClassifier (**A**), (**B**) and (**C**) were obtained from applications to males’ only, males and females, and nulliparous women datasets respectively. Areas under ROC curve (AUC) for each experiment are appended on the corresponding plots. Results were produced from 4-fold cross-validation. The BamClassifier has higher AUC as compared to the random model for each application.

These results further support the performance of BamClassifier as superior for the assessment of ID. In addition, BamClassifier achieved 100% AUC in all the evaluations performed in this study (Fig. 4). These performances are consistent with the intrinsic makeup of the method for exploiting initial bagging techniques to reduce variance at the learning of patterns in the data, while taking advantage of the disparity between the positive and negative labels in assessing ID for enhanced inference. The final stage of consensus predictions of instances improves performance accuracy.

### Stability assessment of BamClassifier

Given the high performance of the proposed model, a further assessment was conducted to evaluate its stability across 100 bootstrap samples generated from the datasets used in the study. The means, standard deviations, and 95% confidence intervals for the model’s performance metrics are presented in Table 5. This evaluation supports the model’s generalization ability on new, unseen data and its sensitivity to variations within the data. Overall, the model demonstrated considerable stability, as reflected by high mean values, low standard deviations, and narrow confidence intervals for the performance metrics (Table 5).

**Table 5:** Performance of BamClassifier in stability assessment.

Metrics	Mean	Standard deviation	95% Confidence interval	
			Lower limit	Upper limit
Males only data				
Accuracy	0.94	0.02	0.91	0.98
Specificity	0.96	0.02	0.92	0.99
Sensitivity	0.86	0.07	0.70	1.00
Precision	0.76	0.09	0.62	0.92
Nulliparous women data				
Accuracy	0.94	0.01	0.92	0.96
Specificity	0.97	0.01	0.95	0.99
Sensitivity	0.89	0.03	0.84	0.94
Precision	0.94	0.02	0.89	0.98
Both males and females data				
Accuracy	0.95	0.02	0.91	0.98
Specificity	0.97	0.01	0.95	0.99
Sensitivity	0.86	0.06	0.76	1.00
Precision	0.85	0.05	0.75	0.96

**Fig. 5:**
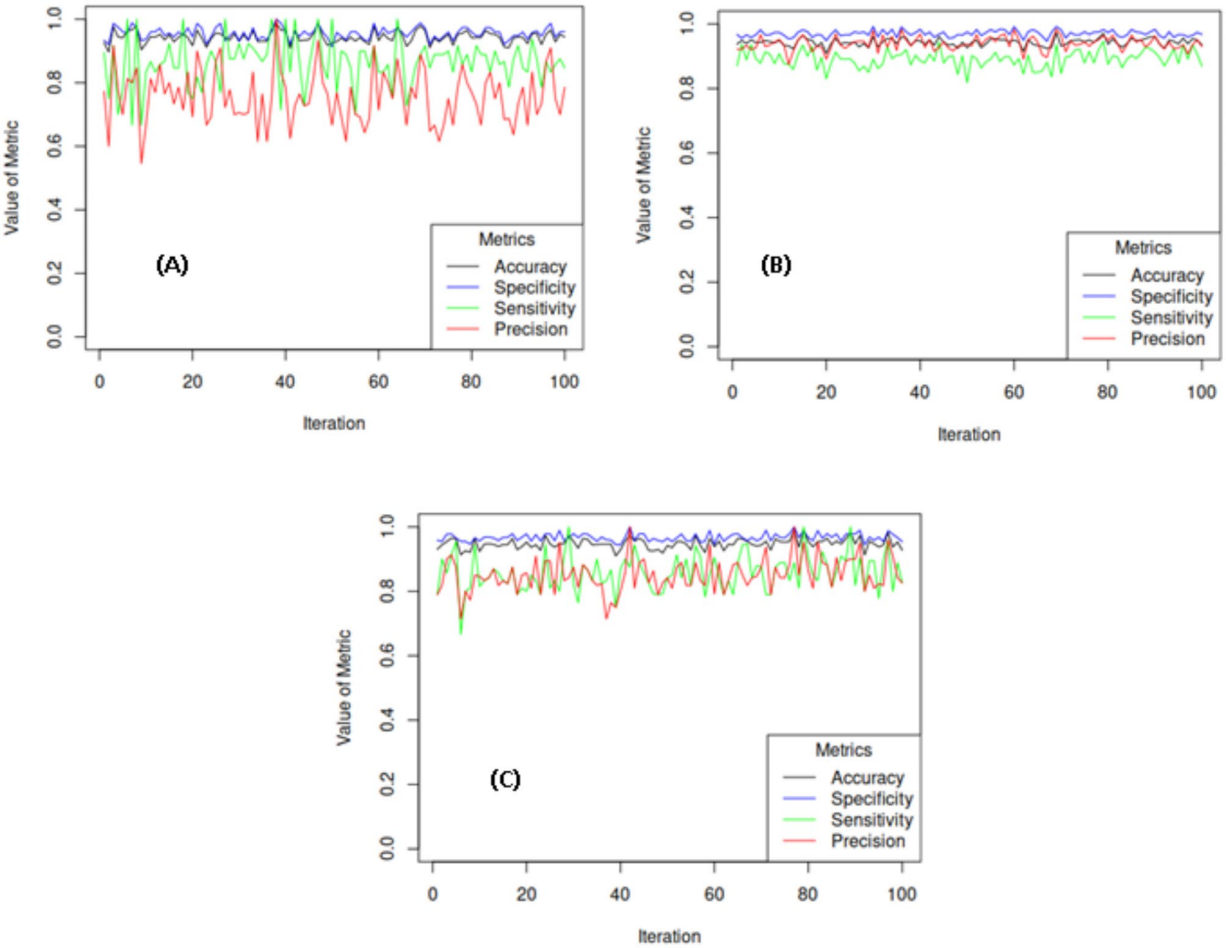
Varying performance of BamClassifier over bootstrap samples. (**A**) Performance on Males’ only data, (**B**) Performance on Nulliparous women data and (**C**) Performance on Both Males and Females’ data.

Additionally, the range of distribution for each performance metric in the experiments was consistent with earlier results, reinforcing the model’s high performance in each iteration across the bootstrap samples (Fig. 5).

### Robust assessment of bamclassifier

In order to further evaluate the robustness of BamClassifier, its performance was systematically assessed under different simulated conditions: class imbalance, feature noise, and varying data distributions. As the original dataset did not follow a standard distribution, these simulations allowed a comprehensive robustness analysis. For a highly imbalanced dataset, the model achieved near-perfect accuracy, specificity, sensitivity, and precision (Table 6), underscoring the consistency of median-supplement methods, particularly when significant differences exist between training data labels^22^. This label distinction amplifies the supplementary data incorporated into the training set, strengthening the machine learning model’s performance. Consequently, the high performance observed in the imbalanced data scenario aligns with expectations of BamClassifiers. The model also maintained high performance in datasets with varying levels of feature noise (Table 6). When training data was simulated data from the Gamma distribution, the model achieved accuracy, specificity, and sensitivity exceeding 99%, with a precision of 97% (Table 6). This high level of performance, without compromising the AUC and diagnostic odds ratio (Table 6), demonstrates that BamClassifier is robust across key performance metrics.

**Table 6:** Results of robust assessment of BamClassifier.

Characteristic of dataset	Accuracy	Specificity	Sensitivity	Precision	Diagnostic Odds Ratio	AUC*
Imbalanced data	0.996	0.997	0.989	0.968	27451.670	1.000
Flat noise (0.1) in first two features	0.996	0.997	0.989	0.968	27451.670	1.000
Featured noise (noise level = 5% of standard deviation of first two features)	0.997	0.998	0.989	0.978	41223.000	1.000
Featured noise (noise level = 5% of standard deviation of half of features)	0.995	0.996	0.989	0.958	20566.000	1.000
Different distribution	0.993	0.994	0.989	0.974	14948.000	1.000

*Area under receiver operating characteristics.

## Discussion

Having been considered as the main cause of anaemia and other medical conditions, under-diagnosed ID for various reasons affects economies, quality of life, and mortality of individuals^35^. As to conclusive outcomes for existing IDs, no single test is noted to be superior in all conditions^36^. Issues such as difficulty in interpreting test outcomes and setting diagnostic thresholds lead to misdiagnosis and lack of agreement among different healthcare providers^37^. To standardize ID assessment outcomes, computational approaches having empirically grounded outcomes provide relatively unambiguous outcomes. In this study, BamClassifier, a new machine learning technique, is introduced to provide accurate and straightforward outcomes in assessing ID. This method combined most of the factors known to be linked to ferritin levels and used in the assessment of IDs in laboratories into a set of features for learning and modelling. The method reverse-engineered models by building patterns based on the features and values for a collection of instances or samples to predict ID assessment outcomes. Multiple tests of the methods on real datasets obtained from Ghana and simulated datasets have shown that the method is very effective resulting in accurate outcomes and has sensitivity that compares favourably with other established methods.

While BamClassifier achieved perfect (100%) AUC for all the experiments (Fig. 4), other methods based on the random forest predicting ferritin concentration could only achieve a maximum AUC of 92% although they outperformed specialists in laboratory medicine^19^. We note that while^19^ considered just complete blood count and C-reactive protein measures from studies in Germany, we included additional attributes for assessment of ID such as HB, MCV, MCH, and MCHC measured from studies in Ghana. Nevertheless, a similar study employing similar attributes that developed logistic regression, artificial neural network and adaptive neuron-fuzzy based models could only achieve as high as 98% AUC from studies conducted in Iran to assess ID anaemia in both males and females^21^. Even though our method was focused on only ID assessment to avoid any confounding effects, it achieved better AUCs to indicate it has better predictive prowess. These perfect AUCs are attributable to the contributing measures of sensitivities and specificities.

Particularly, BamClassifier obtained specificity of 97% each on the experiments involving nulliparous women, and males and females’ dataset, and 96% on the males’ only data where it was outperformed by random forest method (Tables 2 and 3, and Table 4). However, it far outperformed naive Bayes, random trees, logistic regression, Bayesian network and random forest in terms sensitivity, precision, and diagnostic odds ratio in all the experiments conducted in this study. The results suggest that although other machine learning techniques could be used in ID assessment, BamClassifier has superior performance achieving significantly higher correct predictions compared to them.

The difference in accuracy of our methods and the others is amplified in the relatively larger dataset (Nulliparous women’s data). These are suggestive that within this context of application, the computational methods are able to handle the relatively smaller data. Nevertheless, the naive Bayes method was the least performer and this is the result of having predicted the least true positive and negative. However, boosting this in the BamClassifier exploits its capabilities along with bagging methods. While random forest is a well-known improvement of tree-based methods including random trees, it is not always better than other probabilistic-based methods such as Bayes Network and logistic methods as demonstrated on the application to the ID detection task (Fig. 3). These are mostly the cases where attributes have independent observations and are randomly measured as observed in this study.

These findings are further supported by the results of the overall classification rates indicated by the accuracy metric. BamClassifier had the best accuracy in all the experiments, exceeding 94% while the best of the other methods was 86% (Fig. 4). The performances on all the datasets are the results of the method’s ability to find higher numbers of true labels for every test instance compared to the other methods. These true labels, ID positive and negative statuses, account for the true positive and true negative counts that yield the high accuracy, precision, specificity, sensitivity, and diagnostic odds ratio. While ensuring that true labels are assigned in the final stage of the method, it employs a probability in the bag of consensus predictions to ensure that false negative and false positive counts are reduced. The probability-based assignments of labels in the final stage of the method ensure that the highest occurring label for every instance is assigned to it. This explains why BamClassifier has significantly better performance indicated by the DOR exceeding the other methods by hundreds. These performances were consistent in other tasks involving simulated data (Tables 5 and 6). More importantly, these performances were obtained from real practical assessments suggesting their utility for practice.

It has been demonstrated in previous studies regarding the potential of computational approaches to improve ID assessments^21,38^. This study presents significant advancements of these approaches to ID assessments. It uses routine CBC laboratory data to provide accurate and standardized information about ID while it is not invasive. Further, an application of the methods would allow for multiple assessments of samples, saving time and cost. These could lead to earlier and more widespread detection of iron deficiency, facilitating timely interventions and reducing the burden on already strained healthcare infrastructures especially in developing countries such as Ghana.

The proposed method is currently designed for assessment of ID and could be readily applied to other binary classification problems as well, which may limit its applicability to multiclass applications where more distinctions that are complex are needed. Additionally, the proposed method assumes unequal distribution of labels within data, a condition that may not be present in all datasets. This requirement may not be satisfied by other data, which is rarely encountered within real life applications of the study area. To apply the model to other domains beyond its intended purpose, it would require some necessary modifications to generalize across diverse data structures.

## Conclusion

Reliable and accurate assessment of ID is necessary for effective intervention and prevention strategies for associated diseases. Here, we presented a new machine learning technique, BamClassifier, for assessing IDs. This method obtained optimal sensitivity, specificity, precision, DOR, and accuracy compared to other established methods when applied to groups of males, females, and both males and females’ datasets. It was shown that although other methods are applicable to the problem, BamClassifier achieved exceptional performance metric scores, and in addition, obtained perfect AUC scores. These scores were found to be better compared to other applications that had been done in the past that outperformed experts in laboratory medicine. A successful application of the method permits the simultaneous study of several samples while standardizing the interpretation of outcomes of such assessments.

## Electronic supplementary material

Below is the link to the electronic supplementary material.

Supplementary Material 1

Supplementary Material 2

## Data Availability

Data is provided within the supplementary information file.
