# Supplementary material for: BamClassifier: a machine learning method for assessing iron deficiency
Source: Sci Rep. 2025 Sep 2;15:32264. doi: 10.1038/s41598-025-92892-y (PMC12402083; doi:10.1038/s41598-025-92892-y)
Supplement: Supplementary file 1 — Supplementary Material 1 [file 41598_2025_92892_MOESM1_ESM.pdf]

BamClassifier: a machine learning method for assessing iron deficiency

Emmanuel S. Adabor, Patrick Adu, Daniel Adomako Asamoah

Supplementary Material

[1] Code for implementing BamClassifier

```
bagforest = function(d1,ts=NULL){
  Indices = 1:nrow(d1)
  oob_ind = 0
  MSCY = 0
  oob_ind = 0
  B =100
  realvec = 0
  set.seed(10)
  indep_t_sample = ts
  vMScresults = 0
  for (i in 1:B){
    set.seed(i); b1 = sample(Indices,100,replace=F)
    tr_set = d1[b1,]
    oob_set = d1[-b1,]
    MSCmodel = MSclassifier(tr_set, testset=oob_set, method = "MSNaiveBayes")
    vMSCPred = MSclassifier(tr_set, testset=indep_t_sample, method = "MSNaiveBayes")
    oob_ind = c(oob_ind,Indices[-b1])
    MSCY = c(MSCY,as.vector(MSCmodel))
    vMScresults = c(vMScresults,as.vector(vMSCPred))
    realvec = c(realvec,as.vector(oob_set$label))
  }
   MSCY = MSCY[-1]
  vMScresults = vMScresults[-1]
  realvec = realvec[-1]
  oob_ind = oob_ind[-1]

  newY <- vector()
  for (i in 1:length(unique(oob_ind))){
```

```
  tell = which(oob_ind == i)
  klasses = MSCY[tell]
  MFP = which(table(klasses) == max(table(klasses)))

  if (length(MFP) == 1){
    newY[i] = klasses[MFP]
  }
  else
  {
    newY[i] == klasses[MFP[1]]
  }

}
Results = newY
if (!is.null(ts)){
  number_of_rows_of_ind_test_sample = nrow(indep_t_sample)
  vrealvec = rep(as.vector(indep_t_sample$label),B)
  indep_indices = rep(1:number_of_rows_of_ind_test_sample,B)
  test_sample_results <- vector()
  for (i in 1:number_of_rows_of_ind_test_sample){
    vtell = which(indep_indices == i)
    vklasses = vMScresults[vtell]
    vMFP = which(table(vklasses) == max(table(vklasses)))

    if (length(vMFP) == 1){
      test_sample_results[i] = vklasses[vMFP]
    }
    else
    {
      test_sample_results[i] == vklasses[vMFP[1]]
    }

  }
```

```
    Results = test_sample_results
  }

  return(Results)

}
```

[2] Sample code for evaluating BamClassifier

```
source('myMfunction.R')       # The file that contains function bagforest
library(ROCR)
library(MSclassifier)         # Available at https://github.com/esadabor/MSclassifier.git
set.seed(123)
d01 = read.table("dataset1.csv", header=T)
d01 = d01[,-c(1,2)]           # Remove unapplicable attributes
indices <- 1:nrow(d01)
realvec = 0
Col_prob = 0
Y=0
 for (m in 1:4){
  s1 = sample(indices,47,replace=F)
  testset  <- data.frame(d01[s1,])
 trainset <- data.frame(d01[-s1,])
  mmodel <- bagforest(trainset,testset)
  prob_set = rep(2,nrow(testset))
  pre_pro = table(mmodel,testset$label)
   prob_0 = ifelse('0' %in% rownames(pre_pro) && '0' %in% colnames(pre_pro), pre_pro['0', '0'] /
sum(pre_pro), 0)
   prob_1 = ifelse('1' %in% rownames(pre_pro) && '1' %in% colnames(pre_pro), pre_pro['1', '1'] /
sum(pre_pro), 0)
  p0 = which(testset$label == 0)
  prob_set[p0] = prob_0
  prob_set[-p0] = prob_1
  Col_prob = c(Col_prob,prob_set)

  Y = c(Y,mmodel)
  realvec = c(realvec,testset$label)
  indices <- setdiff(indices, s1)
}
Col_prob = Col_prob[-1]
mY = Y[-1]
```

```
evatest = realvec[-1]
pred = prediction(Col_prob,evatest)
roc = performance(pred,"tpr","fpr")
 plot(roc,col="blue", ylab="Sensitivity",xlab="False Positive Rate")
abline(a=0,b=1,col="gray")
negauc = performance(pred,"auc")
AUC = unlist(slot(negauc ,"y.values"))
AUC = round(AUC,2)
legend(0.6,0.2, AUC, title="Area under curve")
eval = performance(pred,"acc")
max_acc = which.max(slot(eval,"y.values")[[1]])
max_acc_value = slot(eval, "y.values")[[1]][max_acc]
optimal_cutoff = slot(eval, "x.values")[[1]][max_acc]
table(evatest,mY >= optimal_cutoff)
```

[3] Code for assessing stability of BamClassifier

```
d01 = read.table("dataset1.csv", header=T)
d01 = d01[,-c(1,2)]
n_points = nrow(d01)
indices <- 1:n_points
Col_prob = 0
Accuracy=0
Specificity = 0
Sensitivity = 0
Precision = 0
DOR = 0
AUC = 0
for (m in 1:100){
  set.seed(m)
  s1 = sample(indices,100,replace=T)
  testset  <- data.frame(d1[-s1,])
  trainset <- data.frame(d1[s1,])
  mmodel <- bagforest(trainset,testset)
  prob_set = rep(2,nrow(testset))
  pre_pro = table(mmodel,testset$label)
  prob_0 = pre_pro['0','0']/sum(pre_pro) # stores probability of pred 0
  prob_1 = pre_pro['1','1']/sum(pre_pro) # stores probability of predicting 1
  p0 = which(testset$label == 0)
  prob_set[p0] = prob_0
  prob_set[-p0] = prob_1
  Col_prob = prob_set
  mY = mmodel
  evatest = testset$label
  pred = prediction(Col_prob,evatest)
  eval = performance(pred,"acc")
  max_acc = which.max(slot(eval,"y.values")[[1]])
  max_acc_value = slot(eval, "y.values")[[1]][max_acc]
  optimal_cutoff = slot(eval, "x.values")[[1]][max_acc]
```

```
  mtable = table(evatest,mY >= optimal_cutoff)
  FN = mtable[1,1]; TP=mtable[1,2]; FP=mtable[2,1]; TN=mtable[2,2]
  Accuracy=c(Accuracy, (TP+TN)/(TP+TN+FP+FN))
  Specificity = c(Specificity, TN/(TN+FP))
  Sensitivity = c(Sensitivity, TP/(TP+FN))
  Precision = c(Precision,TP/(TP+FP))
  DOR = c(DOR, (TP*TN)/(FP*FN))
  negauc = performance(pred,"auc")
  AUC = c(AUC,round(unlist(slot(negauc ,"y.values")),2))
}
Accuracy=Accuracy[-1]
Specificity = Specificity[-1]
Sensitivity = Sensitivity[-1]
Precision = Precision[-1]
DOR = DOR[-1]
AUC = AUC[-1]
Metrics=c('Accuracy','Specificity','Sensitivity','Precision','DOR','AUC')
Sort_Accuracy = sort(Accuracy);
Sort_Specificity = sort(Specificity)
Sort_Sensitivity = sort(Sensitivity)
Sort_Precision = sort(Precision)
Sort_DOR = sort(Precision)
Sort_AUC = sort(AUC)
Acc_lower_limit = Sort_Accuracy[ceiling((2.5/100)*length(Sort_Accuracy))]
Spec_lower_limit = Sort_Specificity[ceiling((2.5/100)*length(Sort_Specificity))]
Sens_lower_limit = Sort_Sensitivity[ceiling((2.5/100)*length(Sort_Sensitivity))]
Prec_lower_limit = Sort_Precision[ceiling((2.5/100)*length(Sort_Precision))]
DOR_lower_limit = Sort_DOR[ceiling((2.5/100)*length(Sort_DOR))]
AUC_lower_limit = Sort_AUC[ceiling((2.5/100)*length(Sort_AUC))]
Acc_Upper_limit = Sort_Accuracy[ceiling((97.5/100)*length(Sort_Accuracy))]
Spec_Upper_limit = Sort_Specificity[ceiling((97.5/100)*length(Sort_Specificity))]
Sens_Upper_limit = Sort_Sensitivity[ceiling((97.5/100)*length(Sort_Sensitivity))]
Prec_Upper_limit = Sort_Precision[ceiling((97.5/100)*length(Sort_Precision))]
DOR_Upper_limit = Sort_DOR[ceiling((97.5/100)*length(Sort_DOR))]
```

```
AUC_Upper_limit = Sort_AUC[ceiling((97.5/100)*length(Sort_AUC))]
```

[5] Additional code for robust assessment of BamClassifier

```
# Simulating imbalanced data
set.seed(123)
n_features <- 8
n_instances <- 188
md1 = read.table("dataset1.csv", header=T)
md1 = md1[,-c(1,2)]
Headingsfdata = names(md1)
original_data <- md1[,-ncol(md1)]
ecdfs <- lapply(original_data, ecdf)
sample_from_edf <- function(ecdf_func, n_samples) {
  u <- runif(n_samples)
  quantile(ecdf_func, probs = u)
}
n_samples <- 1000
X_simulated <- sapply(ecdfs, sample_from_edf, n_samples = n_samples)
class_imbalance_ratio <- 0.90
labels <- sample(c('1', '0'), n_samples, replace = TRUE, prob = c(class_imbalance_ratio, 1 - class_imbalance_ratio))
data_simulated <- data.frame(X_simulated, label = labels)
## Add feature noise
#noise_level <- 0.1  # Moderate noise
#noise_level <- 0.5*sd(X_simulated[,1:2])
noise_level <- 0.05*sd(X_simulated[,1:4])
data_simulated_noisy <- data_simulated
#data_simulated_noisy[, 1:2] <- data_simulated_noisy[, 1:2] + rnorm(n_samples, mean = 0, sd = noise_level)
data_simulated_noisy[, 1:4] <- data_simulated_noisy[, 1:4] + rnorm(n_samples, mean = 0, sd = noise_level)
### Varying distributions
X_skewed <- matrix(rlnorm(n_samples * n_features, meanlog = 0, sdlog = 1), ncol = n_features)
class_imbalance_ratio <- 0.90
labels_skewed <- sample(c("1", "0"), n_samples, replace = TRUE, prob = c(class_imbalance_ratio, 1 - class_imbalance_ratio))
```

```
data_skewed <- data.frame(X_skewed, label = labels_skewed)
```
